# Impact of patient-reported taxane-induced peripheral neuropathy on dose reductions or worsening quality of life in Black women with breast cancer: Analysis from ECOG-ACRIN EAZ171

**DOI:** 10.1002/cncr.70466

**Published:** 2026-06-01

**Authors:** Tarah J. Ballinger, Fengmin Zhao, Guanglong Jiang, Fei Shen, David Cella, John D. Peipert, Kathy D. Miller, Antonio C. Wolff, Angela M. DeMichele, Bryan P. Schneider, Lynne I. Wagner

**Affiliations:** 1 Indiana University Simon Comprehensive Cancer Center, Indianapolis, Indiana, USA; 2 Dana-Farber Cancer Institute ECOG-ACRIN Biostatistics Center, Boston, Massachusetts, USA; 3 Northwestern Feinberg School of Medicine, Chicago, Illinois, USA; 4 University of Birmingham, Edgbaston, Birmingham, UK; 5 Johns Hopkins University/Sidney Kimmel Comprehensive Cancer Center, Boston, Massachusetts, USA; 6 University of Pennsylvania, Philadelphia, Pennsylvania, USA; 7 University of North Carolina, Chapel Hill, North Carolina, USA

**Keywords:** dose delivery, neuropathy, patient-reported outcomes, quality of life, taxanes

## Abstract

**Background:**

Black patients with breast cancer experience higher rates of taxane-induced peripheral neuropathy (TIPN), which may compromise chemotherapy dose delivery and health-related quality of life (HRQOL). Using the prospective ECOG-ACRIN EAZ171 trial, the authors examined whether patient-reported TIPN predicts dose reductions and longer-term declines in HRQOL and physical function.

**Methods:**

EAZ171 enrolled 249 Black patients planned for (neo)adjuvant taxane; 239 with available patient-reported outcome (PRO) data were analyzed. TIPN was assessed during treatment using the Functional Assessment of Cancer Therapy GOG Neurotoxicity scale (FACT-GOG/NTx) (4-item, 11-item, and single items) and PRO-Common Terminology Criteria for Adverse Events numbness/tingling and interference. HRQOL (FACT-G) and physical function (PROMIS PF-10a) were measured through 12 months. Multivariable mixed-effects logistic models evaluated associations between TIPN, taxane dose reduction due to neuropathy, and 12-month HRQOL or physical function decline.

**Results:**

All TIPN PROs were associated with dose reduction, with the FACT-GOG/NTx 4-item subscale showing the strongest association (odds ratio [OR], 10.8; 95% CI, 4.5–25.9). Twelve-month PRO data were available for 140 patients (59%). Neuropathy reported during treatment was not associated with reduced HRQOL or physical function at 12 months; however, persistent neuropathy at 12 months was associated with worse HRQOL (OR, 5.05; 95% CI, 1.33–19.17) and physical function (OR, 5.29; 95% CI, 1.57–17.83).

**Conclusions:**

TIPN was common and strongly predicted taxane dose reduction in Black patients. The FACT-GOG/NTx 4-item subscale performed best, supporting its use in future trials. Although early TIPN did not predict long-term functional decline, persistent neuropathy adversely affected HRQOL, underscoring the importance of early identification and supportive care to improve dose delivery and equity in breast cancer outcomes.

## INTRODUCTION

Black patients with breast cancer experience significantly higher rates of taxane-induced peripheral neuropathy (TIPN), compared to other races.^1,2^ TIPN can be irreversible and lasting, potentially creating longer term disparities in physical functioning or quality of life.^3–5^ In addition, TIPN is the major cause for dose reductions of curative therapy in early-stage breast cancer. Prior work has demonstrated that Black patients who have a dose reduction of taxane therapy have inferior disease-free survival; this association between dose reductions and lower cure rates is not seen in White patients.^6^ Therefore, disparate rates in TIPN incidence are also resulting in inequitable survival outcomes for Black patients with early-stage breast cancer.

Patient-reported outcome measures (PROMs) are the gold standard for reporting subjective experiences related to neuropathy. PROMs provide a way for patients to tell us about their experience, including the impact of neuropathy on functional status. However, despite being impacted most by TIPN, Black patients have been historically underrepresented in trials and thus their experience is underrepresented as well. EAZ171 was a prospective, pragmatic trial focused entirely on Black patients with early-stage breast cancer, providing a novel opportunity to assess Black patients’ experiences with TIPN directly through administering PROMs. EAZ171 set out to prospectively validate germline markers for TIPN, with key secondary comparisons of taxane type (every 3-week docetaxel vs. weekly paclitaxel) on rates of TIPN and dose reductions.^7^ In addition to evaluation of TIPN by physician adjudication, EAZ171 also included PROMs for a patient-centered assessment of this key toxicity providing an opportunity to compare patient and physician report and the impact of patient-reported neuropathy specifically in Black patients.

Acute toxicities that impact dose delivery and irreversible toxicities that impact long-term well-being may be of heightened concern. Given the impact of TIPN on dose reductions and disease outcomes specifically in Black women, dissecting which PROM might predict dose reduction and long-term consequences would be of substantial clinical relevance. We set out to determine whether using PROMs, and which measure, will predict who will be impacted in terms of both (1) dose reductions, and (2) longer-term functioning and health-related quality of life (HRQOL).

## MATERIALS AND METHODS

### Study design

EAZ171 (ClinicalTrials.gov identifier: NCT04001829) was coordinated by the ECOG-ACRIN Cancer Research Group and enrolled 249 patients between June of 2019 and March of 2022.^7^ All participants self-identified as Black or African American and were planned to receive taxane-based chemotherapy with curative intent. The study was nonrandomized; however, chemotherapy dose and schedule were outlined in the study protocol. The primary objective of the parent trial was to demonstrate increased risk of TIPN in association with a high-risk genotype; ultimately, the high-risk germline genotype was associated with higher risk of TIPN but this did not meet statistical significance. Secondary objectives included comparison of TIPN from paclitaxel versus docetaxel. The finding of significantly lower rates of TIPN and fewer dose reductions in the docetaxel arm were presented previously.^7^

The study was approved by the National Cancer Institute (NCI) and participating site institutional review boards. All patients provided informed consent and the study was conducted in accordance with the Declaration of Helsinki.

### PROM collection

Participants completed PROMs on article in English at baseline, the first day of each taxane chemotherapy cycle, at the end of treatment, and 1 year after initiation of taxane therapy. The description and analyses presented here focus on neurotoxicity measures reported during chemotherapy, as well as HRQOL and physical functioning at the 12-month follow-up.

Patient-reported neuropathy was assessed using two PRO-Common Terminology Criteria for Adverse Events (CTCAE) items^8,9^ and the Functional Assessment of Cancer Therapy GOG Neurotoxicity scale (FACT/GOG-NTx).^10^ PRO-CTCAE numbness and tingling (item no. 39) was collected with two questions assessing severity and interference.^11^ PRO-CTCAE attributes were coded 0–4 to describe none, mild, moderate, or severe numbness/tingling or interference from numbness/tingling. Neurotoxicity was assessed using 11 items from the FACT/GOG-Ntx questionnaire. Each item is scored from 0 (not at all) to 4 (very much), and total score is the sum of all 11 items ranging from 0 to 44, with higher numbers indicating less neuropathy.

HRQOL was measured using the sum total score of 27 items from the FACT-General (FACT-G), covering domains of physical, social, emotional, and functional well-being.^12^ Each item is scored from 0 (not at all) to 4 (very much). Total score ranges from 0 to 108, with higher scores indicating better quality of life.

Physical function was assessed using the PROMIS Physical Function Short Form 10a.^13^ Summary scores are calculated as a T score (mean = 50, SD = 10) based on a general population reference, with higher scores reflecting better physical function.

### Statistical analysis

We sought to determine whether patient-reported neuropathy was associated with treatment tolerability, operationally defined as treatment dose reductions or discontinuation due to TIPN. We also examined longer-term detriments to HRQOL and physical function. In addition, we sought to determine which PROM may be most predictive of tolerability-related treatment disruption. Our primary analysis focused on the FACT/GOG-NTx 4 item neuropathy scale, defining TIPN as a decrease of ≥2 compared to baseline. We additionally investigated occurrence of TIPN with alternative definitions including an increase of ≥1 in PRO-CTCAE numbness/tingling interference, a decrease in total FACT/GOG-Ntx 11-item subscale score of ≥4, or an increase of ≥1 in FACT/GOG-Ntx individual items assessing numbness or tingling in hands (item 1) or feet (item 2). These change scores are based on clinically meaningful changes established for the FACT/GOG-Ntx.^14^ For single item measures, we considered an increase in one grade, or one point, to be clinically meaningful.

We evaluated the association of neuropathy by any of the above definitions with subsequent dose reduction of taxane chemotherapy as a dichotomous categorical variable (yes/no). Dose reductions included dose cessations; however, only three patients discontinued therapy, two from TIPN but only after experiencing dose reduction due to TIPN first, and a third from an adverse event that was not neuropathy. Therefore, only dose reductions are included in the analysis. If a patient required multiple dose reductions, this was considered one dose reduction event.

We also evaluated the association of neuropathy by any of the above definitions with subsequent reduction in HRQOL or physical function at 1 year. Reduced HRQOL was defined as a reduction in total FACT-G score of ≥7 and reduced physical function was defined as a reduction in PROMIS physical function T-score of ≥5. These outcome measures of decreased function were assessed as dichotomous categorical variables (yes/no). The score changes used were based on established clinically meaningful difference ranges for these questionnaires.^15^

Multivariate logistic mixed effect regression models with random intercept (repeated measures within patients) were used to assess the associations between occurrence of TIPN and dose reduction. Multivariate logistic regression models were used to assess the association between occurrence of TIPN (during treatment and at 12 months) and reduction in HRQOL and physical function at 12 months. Adjusting covariates included baseline neuropathy score, patient age at registration, body mass index, Eastern Cooperative Oncology Group performance status (ECOG PS), disease stage, nodal status, estrogen receptor status, HER2 status, hemoglobin A_1c_ level, and treatment arm (paclitaxel or docetaxel) in all regression models. No adjustment was made for multiple comparisons and a *p* value of <.05 was considered statistically significant.

## RESULTS

### Follow-up and PRO completion rates

**FIGURE 1:**
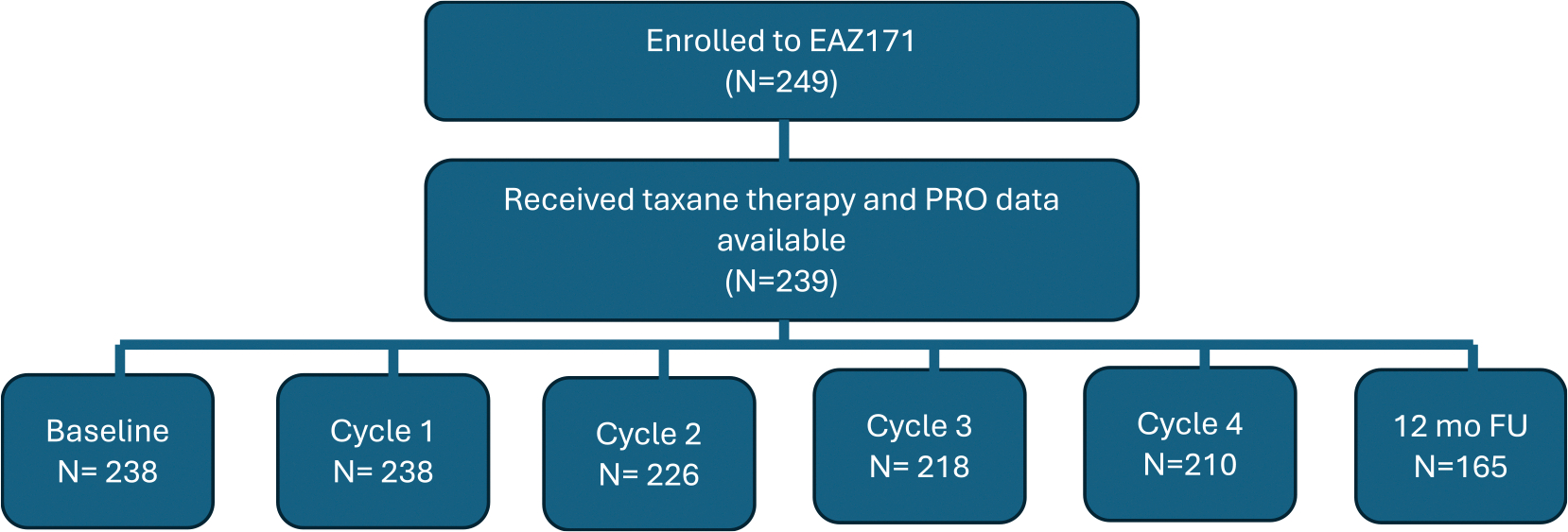
Consolidated Standards of Reporting Trials. Participating patients with patient-reported outcome data.

Of the 249 patients enrolled, 239 received at least one dose of taxane and are included in this analysis. The number of patients providing PRO data at each time point is shown in the Consolidated Standards of Reporting Trials diagram (Figure 1). Of evaluable patients, 226 (94.6%) reached the 1-year time point and 164 patients (68.6%) provided PRO data at 12 months.

### TIPN by FACT/GOG-NTx and PRO-CTCAE

Average scoring for numbness/tingling severity and interference by FACT/GOG-NTx and PRO-CTCAE are shown in Table 1 at each time point. Rates of moderate or severe numbness/tingling by PRO-CTCAE at any point between baseline and 12 months post-registration were 32% (*n* = 53 of 164).

### Comparison of physician adjudicated TIPN and PRO-CTCAE

**FIGURE 2:**
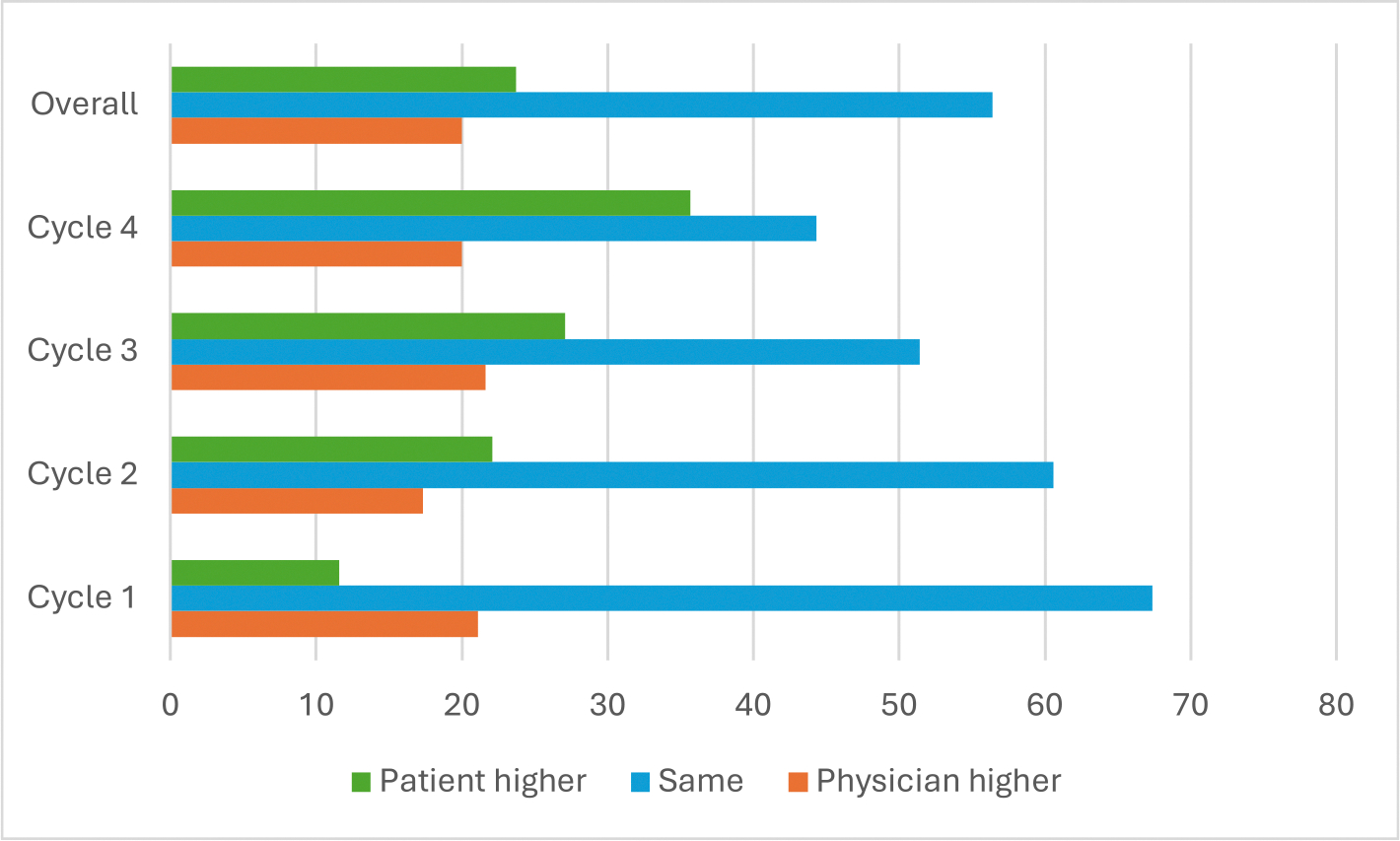
Physician and patient concordance in TIPN severity using CTCAE and PRO-CTCAE. CTCAE indicates Common Terminology Criteria for Adverse Events; PRO, patient-reported outcome; TIPN, taxane-induced peripheral neuropathy.

A comparison of physician and patient grading of neuropathy severity at each cycle was performed and is reported descriptively in Figure 2 for the first four taxane cycles. Overall, agreement was reached in 56% of instances. Physicians reported more severe neuropathy in 20% of instances and patients reported more severe neuropathy in 24% of instances, with the difference being larger at later cycles of taxane therapy.

### Association between PRO measures and incidence of dose reductions due to TIPN

We sought to determine the predictive ability of each PROM to capture subsequent need for dose reductions of taxane therapy due to TIPN. Of 239 patients receiving taxane therapy, 76 (31.8%) required a dose reduction due to TIPN, including 47 (39%) of the patients in the paclitaxel arm and 29 (24%) in the docetaxel arm. Covariates associated with dose reduction included baseline FACT/GOG-NTx score, taxane arm, and ECOG PS. Odds ratio (OR) for dose reduction based on neuropathy symptoms by each PROM in multivariate analysis are shown in Table 2.

The 4-item sensory neuropathy specific subscale of FACT/GOG-NTx had the largest OR for subsequent dose reduction. For the 64.8% of participants reporting a decrease of ≥2 points, there was an increased risk of subsequent dose reduction with an OR of 10.8 (95% CI, 4.5–25.9, *p* < .001). By FACT/GOG-NTx 11-item neuropathy subscale total score, 61.5% had a significant decrease in score of ≥4 points during taxane therapy, indicating worsening neuropathy. This decrease was associated with increased risk of dose reduction (OR, 5.02; 95% CI, 2.55–9.88, *p* < .001). We also investigated single items 1 (numbness or tingling in the hands) and 2 (numbness or tingling in the feet), with higher scores indicating more severe symptoms. As shown in Table 2, both were significant, with the hand item being more predictive (OR, 6.10; 95% CI, 2.96–12.57) than the feet item (OR, 3.88; 95% CI, 2.0–7.5).

Of the participants providing PRO-CTCAE data, 67.8% reported an increase in numbness/tingling severity of at least one point and 49% reported an increase in numbness/tingling interference of at least one point during taxane chemotherapy. On multivariate analysis, an increase in PRO-CTCTAE numbness/tingling severity of ≥1 point was associated with increased risk of subsequent dose reduction (OR, 8.16; 95% CI, 3.65–18.25, *p* < .001), and an increase in numbness/tingling interference of ≥1 point was also associated with significantly increased risk of subsequent dose reduction, although to a lesser degree (OR, 5.64; 95% CI, 2.89–10.98, *p* < .001).

### Association between PRO measures and HRQOL and physical functioning

We further sought to determine the association between PROMs for TIPN during chemotherapy and decreases in HRQOL measured by FACT-G and physical functioning measured by the PROMIS PF Short Form 10a at 1 year, with results shown in Table 3. Of 164 patients with available HRQOL data at 1 year, the average FACT-G total score improved from baseline to 1 year (+4.3; SD, 17.0; *p* < .01) and only 18.9% (31 of 164) of participants had worse quality of life at 1 year. On multivariate analysis, no covariate investigated was associated with a significant decrease in HRQOL at 1 year. Although patient-reported TIPN during treatment by FACT-GOG/NTx 4 item subscale was associated with subsequent reduction inHRQOL on univariate analysis (OR, 4.65; 95% CI, 1.17–18.48, *p* = .03), this was no longer statistically significant after adjusting for other covariates, likely due to small sample size for those variables (Table 3). However, patients who continued to report worsened neuropathy by the FACT/GOG-NTx 4-item subscale at 12 months did have a significant reduction in HRQOL (OR, 5.05; 95% CI, 1.33–19.17, *p* = .02). This was also true for the FACT/GOG-NTx total score and PRO-CTCAE interference.

To determine if dose reductions due to neuropathy may have blunted the impact of neuropathy on HRQOL, we compared patients who received a dose reduction and those who did not. None of the PROMs predicted worsening HRQOL regardless of whether participants received a dose reduction or not. In addition, due to significant attrition by 12 months, we compared whether patients completing the 12 month time point differed in baseline clinical characteristics or HRQOL compared to those who did not complete it. This analysis found no significant differences (Supporting Information).

There was no change in patient-reported physical function from baseline to 12 months in the total study population (PROMIS PF T score −0.2; SD, 9.5; *p* = .79) and 26.4% (43 of 163) had worse physical function at 12 months. None of the PROMs examined for TIPN developing during therapy were associated with a clinically meaningful decrease in PROMIS PF T score from baseline to 1 year of ≥5 points. However, persistent decline in the FACT/GOG-NTx score or increased neuropathy interference by PRO-CTCTAE at 12 months was associated with a significant decline in physical function (OR, 5.29; 95% CI, 1.57–17.83 and OR, 3.52; 95% CI, 1.17–10.60, respectively).

## DISCUSSION

We report PROMs of taxane-induced peripheral neuropathy from the ECOG-ACRIN trial EAZ171 and their subsequent association with dose reductions, HRQOL, and physical functioning. Our findings underscore the value of PRO-collected TIPN by demonstrating the association of patient-reported TIPN and treatment disruption as well as discordance in patient versus physician grading. EAZ171 enrolled only Black patients, who are both significantly more likely to develop TIPN during curative therapy for breast cancer, and to be impacted by TIPN with subsequent dose reductions and a corresponding reduction in survival.^2,6^ EAZ171 was the first trial to prospectively enroll only this higher risk population and thus represents a key data set to provide insight into the unique patient-reported experience of TIPN in Black patients. Whether using FACT/GOG-NTx or PRO-CTCAE, the majority of participants reported some degree of neurotoxicity during treatment, underscoring the prevalence of TIPN in this population. Patients who reported any significant increase in neurotoxicity were at high risk for subsequent dose reduction of curative therapy, confirming neuropathy as a driver of tolerability-related treatment disruption. Although neurotoxicity during treatment did not predict a detriment to physical function or HRQOL in follow-up, persistent symptoms at 1 year were associated with a decline in both HRQOL and physical functioning, depending on the PROM used.

PROMs can be used for individual treatment decisions and at the population level for selection into interventional trials or risk stratification, or as end points in clinical trials. Here, concordance between physician and patient grading was approximately 56%, with patients more likely to report higher symptoms. This discordance is in line with other literature,^16,17^ but is perhaps more surprising given the primary purpose of the clinical trial was toxicity reporting. This suggests physicians likely do not view PROMs when performing their own grading or making decisions about dose adjustments. Trials in which physicians have been prompted with patient-reported toxicity measures before their own grading show higher concordance.^18^ PROMs are the gold standard for capturing the patient’s experience and should be used across trials and clinical programs for assessment of neuropathic toxicity.^19,20^

The number of available measures or metrics for patient-reported neuropathy makes it difficult to compare across studies or to choose the best measure. Clinical utility of a measure is demonstrated when it can be used to help us improve the lives of patients, either through better survival, improved HRQOL, or less toxicity. Here, each of the PROMs and subscales analyzed were associated with a subsequent reduction in taxane dose, which is associated with worse survival in Black patients.^6^ This association was strongest for increases using only the first four questions from the FACT/GOG-NTx; these questions focus only on sensory neuropathy. The 4-item FACT/GOG-NTx subscale has the advantage of being short and easy to administer in either a clinic or trial setting with minimal burden to patient or staff. The subscale has demonstrated reliability and validity largely in gynecologic oncology populations receiving paclitaxel therapy, where symptoms are primarily sensory.^21^ The first four items address “numbness or tingling” in the hands or feet and “discomfort” in the hands and feet, avoiding the remainder of the questions that include assessment of joint pains, weakness, buzzing in the ears, trouble hearing, or trouble walking, which may be nonspecific and completely unrelated to TIPN.

Contrary to what we hypothesized, we did not find an association between patient-reported neurotoxicity during therapy and subsequent reductions in HRQOL at 1 year. In addition, we observed no reduction in HRQOL from baseline to 1 year in the total population, regardless of neuropathy. Several studies have suggested worse HRQOL in Black breast cancer survivors, compared to White.^22,23^ However, when correcting for tumor and treatment characteristics such as need for chemotherapy, these disparities narrow whereas Black patients maintain superior spiritual health and better social support.^24^ Our study is the first to prospectively evaluate PROMs in a group of homogeneous Black breast cancer survivors all receiving chemotherapy for early-stage breast cancer, and it is reassuring that we did not detect measurable reductions in HRQOL. HRQOL was measured using the FACT-G, which is multidimensional and comprised of physical, social, emotional, and functional well-being subscales.^12^ The baseline questionnaires were administered before any chemotherapy; thus, it is possible baseline score reflected a significant amount of anxiety that improved once therapy was completed, which might blunt any impact of TIPN occurring during therapy on HRQOL. Only approximately two-thirds of the study population filled out the FACT-G at 1 year; however, we did not detect a significant difference in the population who dropped off from the total population in terms of clinical characteristics or baseline HRQOL. It is possible that the trial’s primary focus on TIPN led to better attention and management from care teams, or that dose reductions prevented some deterioration in quality of life. To explore this, we did investigate whether patients reporting neurotoxicity by any measure who had a dose reduction of therapy had significant decreases in HRQOL compared to those without a dose reduction and did not find a difference. Further work is needed to disentangle for whom and when a dose reduction of therapy is truly needed to reduce impact on quality of life while balancing a risk for inferior survival.

We also found patient-reported neurotoxicity during taxane therapy did not predict longer-term reductions in physical function. Physical function was assessed using the PROMIS-PF Short Form 10a, with worse function defined as a T score increase of ≥5. This questionnaire assesses activities of daily living including climbing a flight of stairs, bending or stooping, carrying groceries, washing one’s hair, or dressing independently. It is possible this is not sensitive enough to detect changes in function that are attributable to TIPN, such as putting on jewelry or other small discriminatory tasks, walking longer distances, or wearing fashionable shoes, particularly as the average age of participants in EAZ171 was not geriatric (median age, 54 years; range, 23–80 years). Prior work has shown an association between chemotherapy-induced neuropathy and worse physical function or higher rates of falls and disability.^3,4,25^ However, many studies are cross-sectional in nature and thus correlate current neuropathy with worse physical function; this in contrast to our study, which analyzed patient-reported neurotoxicity during therapy and its association with later, persistent detriments to function.

When looking at the 1-year time point, we did find patient-reported TIPN during therapy by to be associated with reductions in HRQOL and physical function. Prospective, longitudinal data assessing objective and subjective measures of neuropathy and its impact on function are needed in order to inform in whom and when to intervene to prevent worse outcomes, such as in the ongoing PATTERN study (NCT05790538). Studies like this should try to be enriched for Black patients and other groups at highest risk for neurotoxicity.

As with other PROM work, analysis is limited by missing data. It is unknown what selection bias may be introduced based on populations who filled out PROMs at each time point, compared to those who did not. However, it is reassuring that the primary reason for missing data was at the institutional, rather than patient level. Additionally, we based clinically significant changes in scores on prior publications; however, the minimal clinically important difference for a PROM or metric is dependent on the situation in which it is deployed. As noted, prior use of these measures has not been specific to Black patients for whom neurotoxicity may be more common or impactful.

In conclusion, we found patients who reported neurotoxicity during chemotherapy were at high risk for a subsequent dose reduction of taxane therapy, but this did not result in a detriment to physical function or overall HRQOL. However, persistent TIPN symptoms at 1 year were associated with worse HRQOL and functioning. These findings support the potential impact of careful evaluation of toxicity, including after therapy is complete. The 4-item subscale of the FACT/GOG-NTx was most significantly associated with subsequent dose reduction; its ease of administration makes this an ideal measure for future trials in this space. We now plan to use the measures identified here to select patients in need of early or ongoing supportive care to reduce TIPN’s impact on dose delivery in Black patients, potentially improving survival and racial equity in breast cancer.

## Supplementary Material

Supplementary Table

SUPPORTING INFORMATION

Additional supporting information can be found online in the Supporting Information section at the end of this article.

## Figures and Tables

**TABLE 1:** TIPN by FACT/GOG-NTx or PRO-CTCAE in ECOG-ACRIN EAZ171.

	PRO-CTCAE neuropathy severity, range: 0–4	PRO-CTCAE neuropathy interference, range: 0–4	FACT/GOG-NTx 11-item total score, range: 0–44	FACT/GOG-NTx 4-item total score, range: 0–16	FACT/GOG NTx item 1, range: 0–4	FACT/GOG-NTx item 2, range: 0–4

Baseline	0.22	0.15	40.95	14.97	3.75	3.74
C2D1	0.68	0.43	38.50	13.62	3.40	3.38
C3D1	0.90	0.65	37.06	12.45	3.03	3.08
C4D1	1.25	0.88	35.31	11.34	2.73	2.81
EOT	1.32	0.96	34.92	10.87	2.69	2.56
6 months	1.11	0.83	35.73	11.45	2.82	2.86
1 year	1.16	0.90	34.50	10.98	2.78	2.61
2 years	1.49	1.16	33.50	10.00	2.44	2.36

*Note*: Table summarizes mean scores on each questionnaire at each time point. Higher numbers indicate worse TIPN severity or interference for PRO-CTCAE. Lower numbers indicate worse TIPN by FACT/GOG-NTx items.

Abbreviations: CTCAE, Common Terminology Criteria for Adverse Events; EOT, end of treatment; FACT/GOG-NTx, Functional Assessment of Cancer Therapy GOG Neurotoxicity scale; PRO, patient-reported outcome; TIPN, taxane-induced peripheral neuropathy.

**TABLE 2:** Occurrence of patient-reported neuropathy and risk of subsequent dose reduction of taxane therapy.

Change in PRO score	Frequency (%)^a^	OR for dose reduction (95% CI)

FACT/GOG-NTx subscale ≥2	155 (64.8)	10.80 (4.50–25.90)
FACT/GOG-Ntx total ≥4	147 (61.5)	5.02 (2.55–9.88)
FACT/GOG-NTx item 1 ≥1	143 (59.8)	6.10 (2.96–12.57)
FACT/GOG-NTx item 2 ≥1	137 (57.3)	3.88 (2.0–7.5)
PRO-CTCAE severity ≥1	162 (67.8)	8.16 (3.65–18.25)
PRO-CTCAE interference ≥1	117 (49.0)	5.64 (2.89–10.98)

Abbreviations: CTCAE, Common Terminology Criteria for Adverse Events; FACT/GOG-NTx, Functional Assessment of Cancer Therapy GOG Neurotoxicity scale; OR, odds ratio; PRO, patient-reported outcome.

a Number of patients who ever had occurrence of neuropathy during the study treatment and follow-up period in the 239 treated patients.

**TABLE 3:** Occurrence of patient-reported neuropathy and physical function and quality of life at 1 year.

Change in PRO score during taxane therapy	OR for worsening HRQOL at 1 year (95% CI)	OR for worsening physical function at 1 year (95% CI)

FACT/GOG-NTx subscale ≥2	2.8 (0.6–13.0)	2.6 (0.6–11.5)
FACT/GOG-NTx total ≥4	1.5 (0.5–4.6)	3.4 (1.1–11.0)
FACT/GOG-NTx item 1 ≥1	1.4 (0.6–3.3)	0.9 (0.4–2.1)
FACT/GOG-NTx item 2 ≥1	1.1 (0.4–2.7)	2.6 (0.8–8.6)
PRO-CTCAE severity ≥1	3.1 (0.8–11.8)	1.55 (0.5–4.8)
PRO-CTCAE interference ≥1	1.3 (0.4–3.6)	1.6 (0.6–4.4)

Abbreviations: CTCAE, Common Terminology Criteria for Adverse Events; FACT/GOG-NTx, Functional Assessment of Cancer Therapy GOG Neurotoxicity scale; HRQOL, health-related quality of life; OR, odds ratio; PRO, patient-reported outcome.

## Data Availability

Data from the NCI-sponsored trials may be requested from the NCI NCTN Data Archive (https://nctn-data-archive.nci.nih.gov). Data requests may also be submitted to the corresponding author, who may assist in coordinating the request from the appropriate sources. The data that support the findings of this study are available on request from the corresponding author. The data are not publicly available due to privacy or ethical restrictions.
