# Supplementary material for: Impact of patient-reported taxane-induced peripheral neuropathy on dose reductions or worsening quality of life in Black women with breast cancer: Analysis from ECOG-ACRIN EAZ171
Source: Cancer. Author manuscript; Available in PMC 2026 Aug 1. (PMC13428459; doi:10.1002/cncr.70466)
Supplement: Supplementary Table [file NIHMS2191021-supplement-Supplementary_Table.docx]

**Supplemental material**

**Supplemental Table 1.** Clinical characteristics of participants without and with 12 month patient-reported outcome (PRO) data

| Variable | Pts without 12-month PRO data | | Pts with 12-month PRO data | |  |
| --- | --- | --- | --- | --- | --- |
|  | N | % | N | % | P-val |
| Age (median, range) | 54(27,77) |  | 54(23,80) |  |  |
| PS |  |  |  |  | 0.4545 |
| 0 | 60 | 70.59% | 123 | 75% |  |
| 1 | 25 | 29.41% | 41 | 25% |  |
| BMI |  |  |  |  | 0.9043 |
| <25 | 9 | 10.59% | 15 | 9.15% |  |
| 25-30 | 18 | 21.18% | 33 | 20.12% |  |
| >30 | 58 | 68.24% | 116 | 70.73% |  |
| ER status |  |  |  |  | 0.1005 |
| Negative | 44 | 51.76% | 67 | 40.85% |  |
| Positive | 41 | 48.24% | 97 | 59.15% |  |
| PgR status |  |  |  |  | 0.0655 |
| Negative | 49 | 57.65% | 75 | 45.73% |  |
| Positive | 35 | 41.18% | 89 | 54.27% |  |
| Indeterminate | 1 | 1.18% | 0 | 0% |  |
| Her2 status |  |  |  |  | 0.659 |
| Negative | 71 | 84.52% | 142 | 86.59% |  |
| Positive | 13 | 15.48% | 22 | 13.41% |  |
| Hemoglobin |  |  |  |  | 0.4905 |
| <6.5 | 60 | 76.92% | 112 | 72.73% |  |
| >=6.5 | 18 | 23.08% | 42 | 27.27% |  |
| TIPN genetic risk group |  |  |  |  | 0.7531 |
| Low risk | 17 | 22.67% | 40 | 24.54% |  |
| High risk | 58 | 77.33% | 123 | 75.46% |  |
| Baseline GP5 |  |  |  |  | 0.0835 |
| 0 | 9 | 12.68% | 13 | 8.44% |  |
| 1 | 5 | 7.04% | 17 | 11.04% |  |
| 2 | 3 | 4.23% | 24 | 15.58% |  |
| 3 | 10 | 14.08% | 23 | 14.94% |  |
| 4 | 44 | 61.97% | 77 | 50% |  |
| Baseline HRQoL score |  |  |  |  |  |
| Mean (SD) | 80.6(18.8) | | 80.9(18.4) | | 0.91 |
| Median(range) | 85.5(17, 108) | | 84.1(29, 107) | | 0.95 |

PS – performance status; BMI – body mass index; TIPN – taxane induced peripheral neuropathy, GP5 – Functional Assessment of Cancer Therapy item, side effect bother; HRQoL – health-related quality of life
